# Quercetin Improves Barrier Properties in Porcine Small Intestine but Not in Peyer’s Patches

**DOI:** 10.3390/ijms25031530

**Published:** 2024-01-26

**Authors:** Valeria Cornelius, Linda Droessler, Salah Amasheh

**Affiliations:** Institute of Veterinary Physiology, School of Veterinary Medicine, Freie Universität Berlin, 14163 Berlin, Germany

**Keywords:** Peyer’s patch, quercetin, tight junction, epithelial barrier, Ussing chamber, claudins

## Abstract

Peyer’s patches (PPs) are part of the gut-associated lymphatic tissue (GALT) and represent the first line of the intestinal immunological defense. They consist of follicles with lymphocytes and an overlying subepithelial dome with dendritic cells and macrophages, and they are covered by the follicle-associated epithelium (FAE). A sealed paracellular pathway in the FAE is crucial for the controlled uptake of luminal antigens. Quercetin is the most abundant plant flavonoid and has a barrier-strengthening effect on tight junctions (TJs), a protein complex that regulates the paracellular pathway. In this study, we aimed to analyze the effect of quercetin on porcine PPs and the surrounding villus epithelium (VE). We incubated both tissue types for 4 h in Ussing chambers, recorded the transepithelial electrical resistance (TEER), and measured the unidirectional tracer flux of [^3^H]-mannitol. Subsequently, we analyzed the expression, protein amount, and localization of three TJ proteins, claudin 1, claudin 2, and claudin 4. In the PPs, we could not detect an effect of quercetin after 4 h, neither on TEER nor on the [^3^H]-mannitol flux. In the VE, quercetin led to a higher TEER value, while the [^3^H]-mannitol flux was unchanged. The pore-forming claudin 2 was decreased while the barrier-forming claudin 4 was increased and the expression was upregulated. Claudin 1 was unchanged and all claudins could be located in the paracellular membrane by immunofluorescence microscopy. Our study shows the barrier-strengthening effect of quercetin in porcine VE by claudin 4 upregulation and a claudin 2 decrease. Moreover, it underlines the different barrier properties of PPs compared to the VE.

## 1. Introduction

The intestinal epithelium is the largest body surface area with contact with the environment and contains one of the biggest parts of the immune system, the gut-associated lymphatic tissue (GALT) [1]. The porcine GALT consists of mesenteric lymph nodes, isolated lymphoid follicles, and Peyer’s patches (PPs). Those PPs are structured into follicular and interfollicular areas. The follicular areas consist of multiple germinal centers with B-lymphocytes covered by subepithelial domes (SEDs) with macrophages and dendritic cells, and the overlying follicle-associated epithelium (FAE). This builds the barrier between the immunological active follicle and the gut lumen [2]. The FAE is a specialized epithelium and differs morphologically and functionally from the surrounding villus epithelium (VE), for example, by having a thinner mucus layer due to the absence of goblet cells. In contrast, the paracellular barrier in the FAE is tighter than in the VE [3,4], and the basal membrane is more porous than in the VE. Moreover, the FAE contains microfold cells (M-cells), which are specialized for phagocytosis and transcytosis [5,6]. These conditions enable the FAE to implement a controlled uptake of large particles from the lumen and transport them to the SED, where they are either tolerated or they lead to an immunological response [7].

The intestinal barrier is important to protect the organism from luminal toxins, microorganisms, antigens, and allergens, and it is built by the mucus produced by goblet cells, the epithelial cells themselves, and the tight junctions (TJs). TJs are an apical transmembrane connection between epithelial cells and form a selectively permeable paracellular barrier. On one hand, they prohibit diffusion through the paracellular pathway, and on the other, they allow organ- and tissue-specific permeability for cations, anions, or water [8]. The TJ is formed by different transmembrane TJ proteins, namely claudins [9,10], and TJ-associated MARVEL proteins (TAMPs) [11], which are linked to the cytoskeleton via scaffolding proteins like zonula occludens proteins 1, 2, and 3 [12,13]. The barrier properties are determined by the composition of those TJ proteins and differ between epithelia and also along the intestinal axis [8]. In mammals, 27 claudins are described [14] and they can not only be barrier-building but also pore-forming, or even both [8]. Claudin 1 is expressed in most tissues and is a barrier-forming claudin [9,15]. Claudin 2 can be found in so-called ‘leaky epithelia’ like the jejunum or the proximal tubule of the kidney [8,16] and builds a pore for water and cations [17]. Claudin 4 is another sealing claudin, expressed in the kidney, the lung, and the intestine, and it seals the paracellular pathway against Na^+^ [8,18]. Moreover, claudin 4 can also mediate chloride permeability in the kidney [19]. Other barrier-building claudins are claudins 3, 5, 11, 14, or 19, while claudins 10a, 10b, 15, and 17 are pore-forming and claudins 7, 8, and 16 are ambiguous [20]. Occludin is a member of the TAMP family and contributes to TJ stability and the barrier function [21,22], and tricellulin, another TAMP, regulates the permeability of macromolecules in tricellular TJs [23]. In the porcine jejunum, the expression of claudins 1, 2, 3, 4, 5, and 8, and occludin is described [3,24]. In porcine PPs, a higher expression of the sealing claudin 4 was shown in our previous study [3], resulting in a stronger barrier function for PPs compared to the surrounding VE. Especially in the immunologically active PPs, a tight paracellular barrier is essential for controlled antigen uptake by M-cells and dendritic cells to minimize the uncontrolled contact of pathogens and antigens with the SED and the germinal center, which could lead to an immunological overreaction or the development of food allergies [25].

The intestinal barrier function of the TJ is affected by multiple local and systemic diseases. Infections with *Vibrio cholera*, *E. coli*, or *Clostridium perfringens* degrade TJ proteins or cause internalization resulting in an increased permeability [26]. Other diseases linked to impaired barrier function are, e.g., inflammatory bowel disease, type 1 diabetes mellitus, HIV/AIDS, or coeliac disease [26,27,28]. In pigs, a reduced intestinal barrier function was observed in infections with porcine epidemic diarrhea virus (PEDV) [29] or *Clostridium difficile* [30]. Also, the early-weaning of piglets leads to increased intestinal permeability; therefore, TJ regulation is of importance in post-weaning diarrhea (PWD), which is a major challenge in pig production [31]. Endogenous cytokines and growth factors play an important role in physiological and pathophysiological TJ regulation; they can increase (e.g., IL-10, TGF-β, EGF) or decrease (IL-4, INF-γ, TNFα) the barrier function [8,26,32]. Furthermore, nutritional factors can influence TJ composition [26], for example, the trace-element zinc [33], the amino acids glutamine and glycine [34,35], or medium-chain fatty acids [36]. Secondary plant compounds are another group of substances with barrier-affecting properties, e.g., cannabidiol, curcumin, kampferol, berberine, or quercetin [26,37,38]. 

Quercetin is one of the most abundant dietary flavonoids present in many different plants like berries, apples, onions, nuts, or herbs [39], and it shows strong antioxidant characteristics [40]. It has a broad range of biological actions including anti-inflammatory, anti-cancerogenic, and anti-viral properties [41]. Regarding the effect of quercetin on barrier function, a strengthening effect of quercetin was observed in Caco-2 cells [42] and rat ileum and colon [43]. 

Our study focused on the effect on the barrier function of the FAE in porcine jejunal PPs and the surrounding VE. Recent studies from our working group revealed not only different barrier properties between the VE and PPs [3], but also a different reaction in incubation challenges with two known barrier effectors, namely caprate and TNFα [44,45]. Using the Ussing chamber technique, we recorded the transepithelial electrical resistance (TEER) and measured the unidirectional tracer flux of [^3^H]–mannitol to investigate possible changes in the barrier function induced by quercetin. Subsequently, the composition and localization of claudins were analyzed via Western blot, qRT-PCR, and immunofluorescence microscopy. 

## 2. Results

### 2.1. Quercetin Increased TEER Values in the VE but Not in PPs 

**Figure 1:**
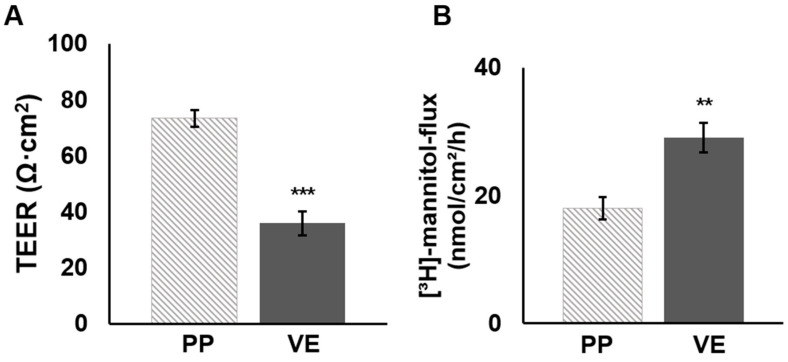
Ussing chamber analysis: Initial comparison revealed stronger functional barrier properties in PPs than in the VE controls. Measurement of (**A**) TEER and (**B**) [^3^H]-mannitol flux rate of PPs and VE prior to incubation with quercetin revealed a higher TEER value (PP: 73.6 ± 1.3 Ω·cm^2^, VE: 33.9 ± 2.4 Ω·cm^2^, *** *p* < 0.001, *n* = 37–38) and a reduced tracer flux of [^3^H]-mannitol is apparent (PP: 17.7 ± 2.3 nmol/cm^2^/h, VE: 29.6 ± 1.3 nmol/cm^2^/h, ** *p* < 0.01, *n* = 12–13).

To analyze the effect of quercetin on porcine PP barrier function, tissue samples of PPs and the VE were mounted into conventional Ussing chambers, and quercetin was added to the mucosal compartment after an equilibration period. We recorded the TEER values and calculated the change to the initial resistance. Before the addition of quercetin, we observed higher TEER values in PPs compared to the VE (PP: 73.4 ± 4.3 Ω·cm^2^, VE: 35.9 ± 2.9 Ω·cm^2^, *p* < 0.0001, *n* = 37–38; Figure 1A) and the reduced unidirectional tracer flux of [^3^H]-mannitol (PP: 18 ± 21.8 nmol/cm^2^/h, VE: 29.1 ± 2.3 nmol/cm^2^/h, *p* = 0.006, *n* = 12–13; Figure 1B). 

**Figure 2:**
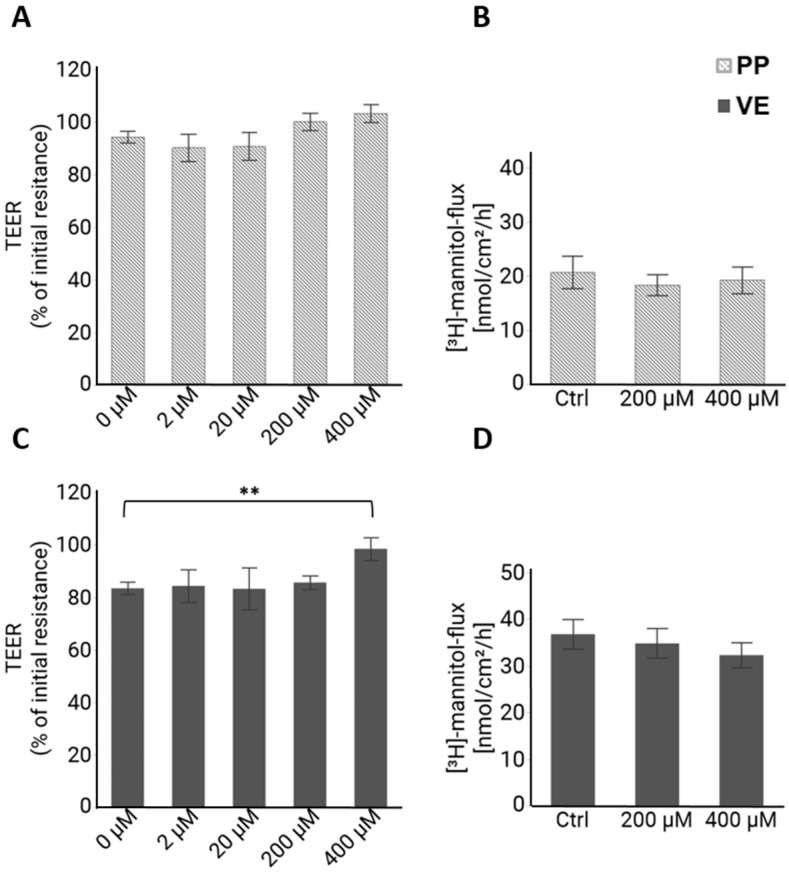
Ussing chamber analysis: TEER and [^3^H]-mannitol flux after 4 h incubation with quercetin. (**A**) TEER of PPs after 4 h of incubation with different concentrations of quercetin as percentage of the initial resistance before quercetin addition. No effect of quercetin on TEER could be observed (*n* = 6–32). (**B**) [^3^H]-mannitol flux from apical to basolateral in PPs after 4 h was not influenced by quercetin (*n* = 13). (**C**) In the VE, 400 µM concentration led to a significantly higher TEER compared to the control (*p* = 0.006, *n* = 31–32). (**D**) [^3^H]-mannitol flux in the VE was not changed (*n* = 13). Values are given as mean ± SEM and asterisks indicate significant difference, ** = *p* ≤ 0.01.

Quercetin was added apically, and after 4 h, no significant effect on TEER could be observed in PP tissue (Figure 2A) compared to the controls. In the VE, incubation with 400 µM of quercetin for 4 h led to a significantly higher TEER value compared to the control group (F (4,92) = 3.28, *p* = 0.015; 0 µM: 83.4 ± 3.3%; 400 µM: 98.5 ± 3.2%, *p* = 0.006, *n* = 31–32; Figure 2C), indicating a barrier-strengthening effect by quercetin. Another indicator of the barrier function is the flux of a tracer molecule, which is not actively transported by intestinal cells. We used [^3^H]-mannitol as a unidirectional tracer, and for both tissue types, the flux was not influenced by 400 µM quercetin (*n* = 13; Figure 2B,D). We had not expected an effect on the paracellular flux in PPs due to the unchanged TEER values; however, the unchanged flux in the VE despite the changed TEER was surprising and will be discussed later. 

### 2.2. Densitometry of Western Blots Revealed Altered TJ Protein Levels by Quercetin in the VE

**Figure 3:**
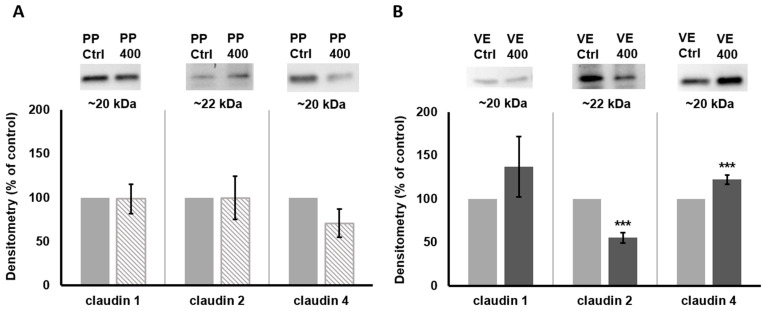
Quantitative protein analysis: Western blots and densitometry of claudins detected in (**A**) PPs and (**B**) VE tissue samples after 4 h incubation with 400 µM quercetin compared to controls. The level of claudin 1 was not influenced by quercetin in both tissue types. For claudin 2, we observed a reduced protein level in the VE (*p* < 0.001) whereas claudin 4 was increased (*p* = 0.001). Values are given as mean ± SEM and asterisks indicate significant differences, *** = *p* ≤ 0.001; protein level is shown as the percentage of controls, set to 100%.

To investigate if quercetin led to a changed TJ composition, we quantified different TJ proteins after 4 h of incubation with 400 µM quercetin (Figure 3). The densitometry of those proteins revealed no effect in PP samples (claudin 1 (98.6 ± 16.9%, *p* = 0.93, *n* = 10), claudin 2 (99.7 ± 24.7%, *n* = 7, *p* = 0.99), and claudin 4 (70.9 ± 16.2%, *p* = 0.08, *n* = 6)). In the VE, sealing claudin 1 was also unchanged (137 ± 35.0%, *p* = 0.30, *n* = 12). For claudin 2, we detected a remarkable decrease to 55.2 ± 5.9% (*p* < 0.001, *n* = 7). Furthermore, claudin 4 was increased to 122.2 ± 5.6%, *p* = 0.001, *n* = 6). A reduction in pore-forming claudin 2 leads to an increased barrier function for ions and therefore to an increased TEER, just like an increase in sealing claudin 4. So, both findings are in accordance with our Ussing chamber results. 

### 2.3. The qRT-PCR Analysis Revealed an Upregulation of Claudin 4 by Quercetin in the VE 

**Figure 4:**
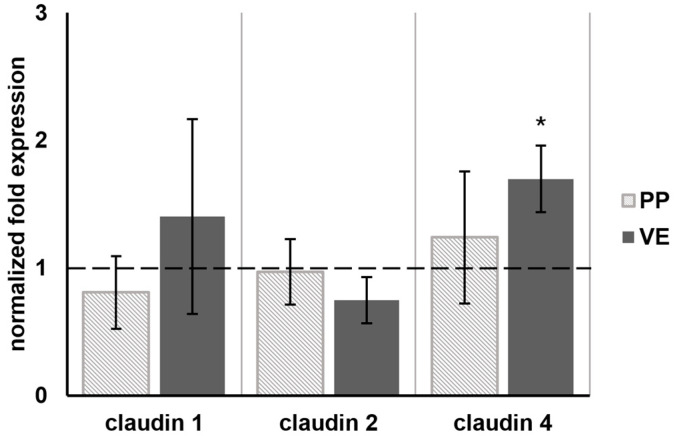
The qRT-PCR: Normalized fold expression of different tight junction protein mRNA after 4 h incubation with 400 µM quercetin. The qRT-PCR revealed an increased mRNA level of claudin 4 in the VE to 1.70 (±0.26, *n* = 4). No effect on the other tight junction proteins could be detected. In PPs, none of the tight junction proteins were influenced by quercetin (*n* = 4). The dashed line indicates the controls. Values are given as mean ± SEM and asterisks indicate significant differences, * = *p* ≤ 0.05.

In addition to the TJ protein amount, we analyzed the mRNA levels of different tight junction proteins. The qRT-PCR revealed no change in the mRNA levels of claudins 1, 2, and 4 in PP tissue samples (*n* = 4) after 4 h of incubation with 400 µM quercetin. In the VE, the expression of claudin 4 was increased to 1.70 (±0.26, *n* = 4; Figure 4 and Table S1). Claudins 1 and 2 were unchanged. 

### 2.4. Immunofluorescence Microscopy Localized Claudins 1, 2, and 4 in the Paracellular Tight Junction Complexes

**Figure 5:**
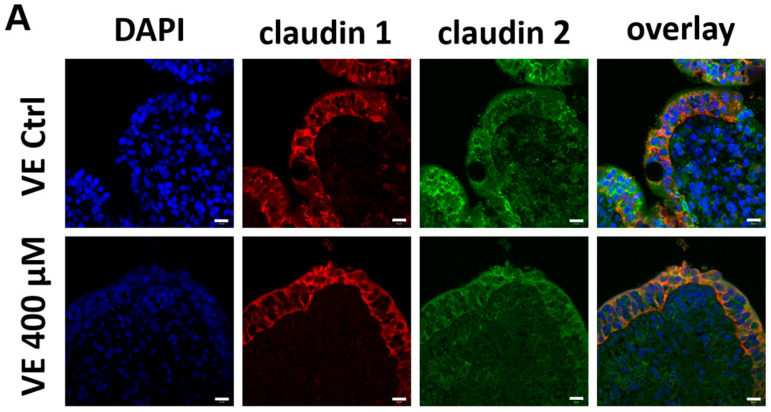

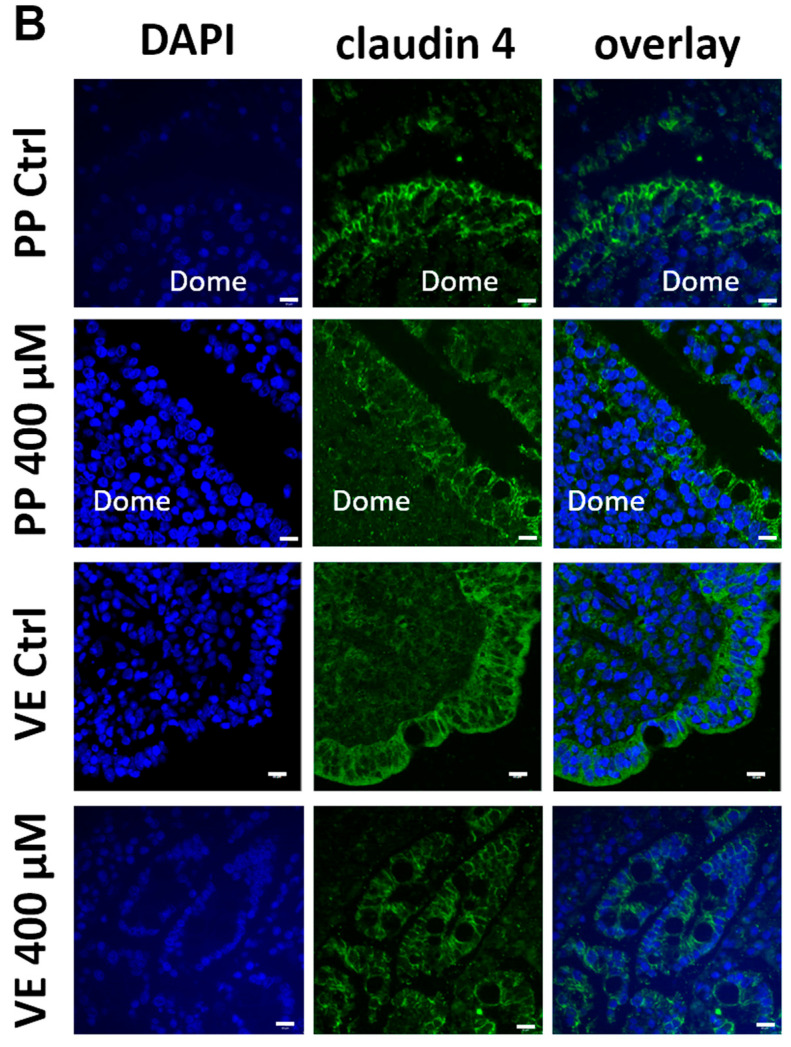
Immunohistochemistry: Immunolocalization of tight junction proteins after 4 h incubation with or without 400 µM quercetin. (**A**) Confocal laser-scanning microscopy of claudin 1 (red) and claudin 2 (green) in the VE samples. (**B**) Widefield microscopy (PP Ctrl and VE 400 µM) and confocal laser-scanning microscopy (PPs 400 µM and VE Ctrl) of claudin 4 (green) in PPs and VE samples. The tight junction proteins can be detected as paracellular signals in the surface epithelium. Cell nuclei were stained blue with DAPI (scale bar: 10 µm, *n* = 5, representative images).

Besides the quantity of tight junction proteins, the localization inside the intestinal cell plays an important role in the function of the protein. Therefore, we performed immunohistological stainings of claudins 1 and 2 in the VE (Figure 5A), and claudin 4 in PPs and the VE (Figure 5B). All three claudins can be localized in the paracellular membrane of the intestinal VE and claudin 4 in the FAE. Due to the autofluorescent background signals caused by the fixation of the tissue samples with PFA and different microscopy techniques (widefield vs. confocal laser scanning), quantification of the claudin signals in our immunohistochemical analysis was not possible; however, this was not intended.

### 2.5. TEER Measurements Revealed No Effects of Quercetin on IPEC J2 Barrier Function

**Figure 6:**
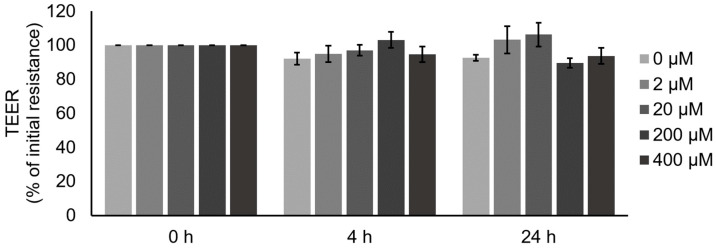
Measurement of quercetin effects on barrier function of IPEC J2 cells: TEER of IPEC J2 monolayers after incubation with different quercetin concentrations. No effect could be observed (*n* = 6–10).

Additionally, we performed incubation experiments with different quercetin concentrations with IPEC J2 cells, an in vitro model of VE. After 4 h incubation with different quercetin concentrations, no effect on TEER could be observed. In addition, after prolonged incubation for 24 h, no effect could be detected (Figure 6; *n* = 6–10).

## 3. Discussion

This study demonstrates the different effects of quercetin on PPs and the VE in the porcine small intestine. The barrier-strengthening effect of quercetin on the VE-like epithelial cell culture model Caco-2 has been described as resulting in a higher claudin 4 protein level [42]. We could show the same effect of quercetin on the porcine VE ex vivo with our Ussing chamber experiment and the subsequent analysis of tight junction proteins. After 4 h of incubation with 400 µM quercetin, the TEER value was significantly higher than in the control group, and an upregulation of claudin 4 mRNA and a higher claudin 4 total protein amount was observed. Claudin 4 is a barrier-forming claudin and the upregulation of claudin 4 results in a tighter paracellular barrier [18]; thus, the upregulation of claudin 4 in our trial is in accordance with the higher TEER value. Besides the quantity of TJ proteins, the localization in the paracellular membrane is critical for the functionality of TJ proteins. Therefore, we performed immunohistological staining of claudin 4, and by employing immunofluorescence microscopy, we visualized the paracellular localization of claudin 4 in quercetin-treated VE samples and also in the controls. 

Another sealing claudin is claudin 1 [8]. In Caco-2 cells, claudin 1 was increased in the detergent-soluble protein fraction in the experiments of Suzuki et al. [42]. In our study, the claudin 1 quantity and localization were unaltered by quercetin. 

In contrast to claudins 1 and 4, claudin 2 is pore forming, permitting paracellular permeability for cations and water. An increased claudin 2 expression results in a decreased TEER and increased paracellular permeability, and this can be observed in different intestinal diseases like IBD or celiac disease [46,47,48,49]. In the VE, we observed a decreased total protein amount of claudin 2, which was located in the TJs. However, this decrease could be due to the degradation of claudin 2 proteins and must not necessarily be linked to a detectable regulation on the transcriptional level, as no significant change was observed on mRNA level. Those findings explain the higher TEER value we recorded after quercetin incubation. 

Besides the TEER, the paracellular barrier properties can also be investigated by the measurement of unidirectional tracer flux. Whereas the TEER reflects the barrier against the movement of ions and solutes across the epithelium [50], unidirectional tracer flux provides information about the permeability of molecules [51]. In our study, we measured to flux of [^3^H]-mannitol, which was unchanged by quercetin incubation in the VE. Due to the higher TEER, an unchanged flux might seem contradictory; however, this is a known effect [45,52], reasoned by two different TJ pathways, namely, the pore and leak pathways. The pore pathway is charge selective and has a junctional diameter of ~6 Å [50]. Larger molecules pass the TJs by the leak pathway. The pore, which is formed by claudin 2, allows the paracellular passage of water and cations [17], but not of larger molecules [53,54,55]. By overexpression of claudin 2 in MDCK cells, Amasheh et al. demonstrated, that the [^3^H]-mannitol flux was not affected; hence, a reduced claudin 2 level also has no effect on the [^3^H]-mannitol flux, which is in accordance with our results. Moreover, Van Itallie et al. overexpressed claudin 4 in MDCK cells and observed an increased TEER value but no effect on the [^3^H]-mannitol flux [18]. Regarding these findings, another paracellular tracer should be considered for future investigations on claudin-2- and also on claudin-4-related barrier effects.

The aim of this study was not only to investigate the effect of quercetin on porcine jejunal VE but also on PP tissue. PPs plays a special role in the immunological defense and have stronger barrier properties than the surrounding VE [3]. In accordance with the results of Radloff et al. [3], we recorded a higher TEER value in PPs compared to the VE and a reduced paracellular flux before the incubation was started. In our study, 400 µM quercetin did not affect barrier properties in PP tissue samples after a 4 h incubation time. Consequently, no effects on the TJ protein mRNA level or total protein amount were detected. The different susceptibility of the VE and PPs against barrier effects was also observed for TNFα by Droessler et al. [45], where a barrier-weakening effect of TNFα on PPs was observed, whereas the VE remained unchanged. In our study, we observed the opposite, which underlines the functional differences regarding the barrier properties in the FAE and its influenceability by barrier effectors.

To underline the barrier-strengthening effect of quercetin in the VE, we performed additional incubation experiments with different quercetin concentrations in an in vitro model of VE, namely IPEC J2 cells. IPEC J2 is a non-transformed cell line that stems from the porcine jejunal epithelium and is used as a model of the small intestine for pigs, but also humans [56,57,58]. After 4 h, the period of the ex vivo Ussing chamber experiments, no effect of quercetin could be detected. We prolonged the incubation for up to 24 h but could not detect any effect either. This might be attributional to the lack of claudin 2 expression in IPEC J2 cells [56]. 

Besides the investigations of quercetin under physiological conditions, like in our experiments or the experiments in Caco-2 cells [42], different studies on the effect of quercetin on the intestinal barrier function under challenged conditions are described. This includes in vitro and ex vivo approaches as well as feeding trials. In HT29/B6 cells, no effect of quercetin on TEER could be observed, but quercetin led to a reduction in claudin 2 and in an incubation challenge with TNFα, and quercetin had a protective effect on the TJs [43]. In the same study, rat colon was incubated with quercetin in Ussing chambers. Quercetin alone increased the TEER, and TNFα and IFN-γ reduced it. Furthermore, in a combined incubation of quercetin and TNFα, quercetin was able to mitigate the barrier-weakening effect of TNFα. Also, in IPEC J2 cells, different incubation challenges were performed and showed a barrier-protective effect of quercetin against barrier perturbation by, e.g., lipopolysaccharide (LPS) [59] or deoxynivalenol (DON) [60,61]. In contrast to our IPEC J2 results, Pomothy [60] and Li et al. [61] observed an increased TEER due to quercetin incubation without any challenge. Whereas both used IPEC J2 cells cultivated with fetal bovine serum (FBS), we used the improved IPEC J2 model by Zakrzewski et al. [56]. In this model, the cells were cultured in porcine serum (PS), and therefore, met more physiological properties of the porcine jejunal tissue. In our previous study focusing on the effects of the plant alkaloid berberine on IPEC J2 cells, similar discrepancies were observed, and we assume a different susceptibility between IPEC J2 cells cultured in FBS or PS [62]. This question needs to be addressed in further research. Nevertheless, Vergauwen et al. also did not observe the effects of quercetin on IPEC J2 cells, despite the usage of FBS for cell cultivation [63]. 

In feeding trials with pigs, the barrier-protective effect of quercetin was shown after weaning [24], DON exposure [61], and transport stress [64].

The transport stress indicates one limiting factor in our approach. To reduce animal trials, we took our tissue samples from a conventional slaughterhouse. We do not know the diet of the pigs, their intestinal health status, nor the stress level prior to slaughtering. Due to the high abundance of quercetin in many plants, we cannot exclude that it was present in the porcine diet. 

With this study, we could underline the different barrier properties and susceptibilities of PPs and the surrounding VE. While a higher sensitivity of the PPs regarding an endogenous substance like TNFα was shown in our previous study, the exogenous quercetin was not able to influence the PP TJs. Quercetin induced an upregulation of claudin 4 in the VE, which is more highly expressed in PPs in general; this could contribute to the lower susceptibility of PPs against quercetin. For a more detailed investigation regarding the different reactions in PPs and the VE, an incubation challenge with TNFα, DON, or LPS combined with quercetin should be considered.

In the VE, we observed a barrier-strengthening effect of quercetin not only by the upregulation of claudin 4 but also by the downregulation of claudin 2. While claudin 4 was increased to 122.2 ± 5.6% (*p* = 0.001, *n* = 6), claudin 2 was remarkably decreased to 55.2 ± 5.9% (*p* < 0.001, *n* = 7). Due to the unchanged [^3^H]-mannitol flux and no effect on claudin-2-deficient IPEC J2 cells, we hypothesize that claudin 2 reduction plays a bigger role in the changed TJ permeability than claudin 4 in the porcine VE, which should be addressed in an improved experimental setup. This could include other flux tracers and incubation challenges with different barrier effectors. 

## 4. Materials and Methods

### 4.1. Ussing Chamber Experiments

#### 4.1.1. Electrophysiology

Pigs were slaughtered at a conventional slaughterhouse and pieces of the distal small intestine were prepared directly afterward by stripping of the outer muscle layers and rinsing the samples carefully with a 4−8 °C cold buffer solution containing (in mmol·L^−1^): Na^+^: 149.9, Cl^−^: 128.8, K^+^: 5, Ca^2+^: 1.2, Mg^2+^: 1.2, HCO^3−^: 25, H_2_PO_4_^2−^: 0.6, HPO_4_^−^: 1.2, and D-glucose: 10 (all from Carl Roth GmbH, Karlsruhe, Germany) at a pH of 7.4. The specimens were placed in a fresh transport buffer and transported to the lab on ice. Normal villus epithelium (VE) and Peyer’s patches (PPs) were used for the experiments and mounted into conventional Ussing chambers with a total buffer exposed area of 0.96 cm². After an equilibration period, different concentrations of quercetin (Sigma Aldrich, Munich, Germany) dissolved in DMSO (Sigma Aldrich, Munich, Germany) were added to the apical side, resulting in a final concentration of 20 µM, 200 µM, or 400 µM. DMSO concentration was 0.1% for all conditions including the control. The buffer was constantly gassed with 95% O_2_ and 5% CO_2_, resulting in a pH of 7.4, and the temperature was maintained at 37 °C. Electrophysiological measurements were obtained by means of a microcomputer-controlled voltage/current clamp (version 9.01 supplied by Dipl.-Ing. K. Mußler, Aachen, Germany). The transepithelial electrical resistance R_t_ [Ω·cm^2^] (TEER) and the short-circuit current (Isc) were continuously recorded. For better comparison, TEER values were calculated relative to the initial resistance before quercetin addition, which was set to 100%. Tissue samples with less than 60% of the initial resistance after 4 h were excluded from further analysis. After 4 h, the incubation and measurements were stopped, and the specimens were taken out of the Ussing chamber and prepared for subsequent analysis of tight junction proteins. 

#### 4.1.2. Paracellular Flux 

In a modified Ussing chamber setup, additional TEER measurements of unidirectional tracer flux from the apical to the basolateral side were performed under short-circuit conditions with [^3^H]–mannitol (PerkinElmer, Waltham, MA, USA). A total of 1 mM non-labeled mannitol (Carl Roth GmbH, Karlsruhe, Germany) was added to the experimental buffer and only the higher quercetin concentrations (200 µm and 400 µM) were used. A total of 3 µCi [^3^H]–mannitol was added to the apical side, and samples from the donor (apical) side were taken directly after the addition of the tracer and at the end of the incubation period. For calculation of the specific activity, the mean was used.

$$Specificactivity\left[nmol\right]=\frac{mean\left({counts}_{donor\;side}\right)}{{concentration}_{donor\;side}\times {volume}_{donor\;side}}$$

Samples from the basolateral side were taken directly before incubation was started and every 60 min during the incubation period. The volume taken was replaced with fresh buffer solution. After the addition of an Aquasafe 300plus liquid scintillation cocktail (Zinsser Analytics, Frankfurt, Germany), the samples were analyzed with a TriCarb 4910TR liquid scintillation counter (PerkinElmer, Waltham, MA, USA). The paracellular flux was calculated with the following equation:

$$J\left[nmol\times {cm}^{-2}\times h^{-1}\right]=\frac{{counts}_{t}\times \frac{V_{chamber}}{V_{sample}}-{counts}_{t-1}\times \frac{V_{chamber}-V_{dilution}}{V_{sample}}}{specificactivity\times area\times time}$$

### 4.2. Tight Junction Protein Analysis

#### 4.2.1. Protein Extraction and Quantification

The tissue samples were taken out of the Ussing chamber and each was cut into two halves; the sample for protein analysis was snap-frozen in liquid nitrogen and stored at −80 °C until further processing. For protein extraction, these samples were homogenized in a Tris-buffer containing, in mmol·L^−1^: Tris (10), NaCl (150), Triton X-100 (0.5), SDS (0.1), and enzymatic protease inhibitors (Complete, Roche, Mannheim, Germany). After homogenization, samples were centrifuged for 1 min at 13,000× *g* (Thermo-Scientific Multifuge X1R, Fisher Scientific, Schwerte, Germany). The supernatant was then cooled on ice for 30 min and retrieved after a second centrifugation step for 15 min at 15,000× *g* at 4 °C (Thermo-Scientific Multifuge X1R, Fisher Scientific, Schwerte, Germany). Protein quantification was carried out using the Pierce reagent (Bio-Rad Laboratories GmbH, Munich, Germany) as instructed, and the EnSpire Multimode Plate Reader (PerkinElmer, Waltham, MA, USA) was used for detection.

#### 4.2.2. Immunoblotting and Densitometry

A total of 10 µg protein and Laemmli buffer (Bio-Rad Laboratories GmbH, Munich, Germany) were mixed and loaded onto a 10% TGX Stain-Free FastCast gel (Bio-Rad Laboratories GmbH, Munich, Germany). After the electrophoresis at 150 V for 60 min, the proteins were transferred to a PVDF membrane (Bio-Rad Laboratories GmbH, Munich, Germany) for 90 min at 100 V. The membrane was blocked for 60 min in 5% milk (in Tris-buffered saline with 0.1% Tween 20) and incubated with the primary antibodies shown in Table 1 overnight at 4 °C. Horseradish-peroxidase-conjugated secondary antibodies raised against mouse or rabbit cells (Cell Signaling Technology, Frankfurt, Germany) were incubated for 1 h at room temperature. After the detection of the total protein amount with the ChemiDoc MP Luminescence imager, Clarity Western ECL Blotting Substrate (Bio-Rad Laboratories GmbH, Munich, Germany) was used to visualize the protein bands. Signals were normalized to the total protein amount and were expressed relative to the control values, which were set to 100%, as described previously [38,65]. Whole Western blot membranes and the corresponding total protein amount are shown in Figure S1. 

### 4.3. RNA Isolation and qRT-PCR

For the qRT-PCR, we used tissue samples of the VE and PPs, each from the control group and the 400 µM group, after 4 h of incubation. Subsequent to the removal and bisecting of the tissue samples from the Ussing chamber, the piece for RNA analysis was transferred into RNAlater (Sigma-Aldrich, Munich, Germany), and stored at −20 °C until total RNA isolation. The isolation was performed with the NucleoSpin RNA II Kit (Macherey-Nagel GmbH & Co. KG, Düren, Germany) as instructed with an additional phenol–chloroform extraction (Carl Roth GmbH, Karlsruhe, Germany) after the homogenization. The RNA concentration was spectrophotometrically quantified (Implen NanoPhotometer P330-30, Munich, Germany), and its quality was determined (2100 Bioanalyzer; Agilent Technologies, Böblingen, Germany). Only samples with an RNA integrity number (RIN) above 6 were included in the experiments. For each sample, the RNA was reverse transcribed to cDNA with the iScript cDNA Synthesis Kit (Bio-Rad Laboratories GmbH, Munich, Germany) according to the manufacturer’s instructions. The synthesis kit (iScript cDNA) was used for first-strand cDNA synthesis with the following protocol: 5 min at 25 °C, 30 min at 42 °C, 5 min at 85 °C, and holding at 4 °C (Mastercycler nexus gradient, Eppendorf, Hamburg, Germany). Transcription reactions without reverse transcriptase were performed to check for possible DNA contamination. Quantitative real-time PCR was performed in the iCycler iQ Real-Time PCR Detection System (Bio-Rad Laboratories GmbH, Munich, Germany) using SYBR green I detection. The reactions were performed as triplicates; the final volume (15 μL) contained iQ SYBR Green Supermix (Bio-Rad Laboratories GmbH, Munich, Germany), primers (0.38 pmol/μL each), and 5 μL cDNA. Detailed information about the primers used is shown in Table 2. Primers were obtained from Eurofins MWG Synthesis GmbH (Ebersberg, Germany). For normalization, the geometric means of the reference genes (beta Actin, GAPDH and YWAHZ) were used and the normalized fold expression was calculated by the ΔΔCt method. 

### 4.4. Immunofluorescence 

The tissue samples were taken out of the Ussing chamber and placed into 4% paraformaldehyde (PFA) (Roti-Histofix, Karlsruhe, Germany) for 24 h. Afterward, an increasing alcohol gradient followed by xylol and paraffin was used for dehydration and paraffin embedding. The paraffin blocks were cut into 5 µm thick sections on a Leica RM 2245 microtome (Leica Microsystems, Heidelberg, Germany). Before the staining step, the sections on slides were deparaffinized in xylol and taken through a decreasing alcohol gradient. Depending on the protein, EDTA or citrate buffer was used for heat-induced antigen retrieval (45 min). After permeabilization of the samples with 0.5% Triton X-100 and blocking with 5% goat serum in PBS, the samples were incubated with primary antibodies raised against claudin 1, claudin 2, and claudin 4 for 60 min at 37 °C or overnight at 4 °C. After a washing step in blocking solution, samples were incubated with goat anti-rabbit Alexa Fluor-488 and goat anti-mouse Alexa Fluor-594 (1:1000, Life Technologies, Carlsbad, CA, USA) and DAPI (1:5000) for 60 min at 37 °C. Sections were then mounted with ProTags Mount Fluor (Biocyc, Luckenwalde, Germany) and analyzed and visualized using a Zeiss 710 confocal laser scanning microscope (Zeiss, Oberkochen, Germany) or a Leica microscope of the DMI 6000 series (Leica Microsystems, Heidelberg, Germany). 

### 4.5. Cell Culturing and Experiments

IPEC J2 is a non-transformed, porcine, intestinal epithelial cell line and was obtained from the DSMZ. Routinely, the cells were cultured in Dulbecco’s MEM/Ham’s F-12 (Biochrom, Berlin, Germany), supplemented with 10% porcine serum (Sigma Aldrich, Munich, Germany) and 1% penicillin–streptomycin (Sigma Aldrich, Munich, Germany) at 37 °C in a humidified 5% CO_2_ atmosphere. The medium was changed every 2–3 days and the cells were split once a week. Experiments were carried out with cells between passages 8 and 15. Therefore, the cells were seeded on semipermeable PCF-culture plate inserts with a size of 12 mm and a pore diameter of 0.45 µm (Millipore, Darmstadt, Germany), which were placed in 12-well cell culture plates. The transepithelial electrical resistance (TEER) was measured with a chopstick electrode and an epithelial volt–ohm meter (EVOM) (World Precision Instruments, Sarasota, FL, USA) every 2–3 days, beginning after 10 days. As soon as the TEER values were stable (~14 d), incubation with quercetin was started. Quercetin stock solution, dissolved in DMSO, was prepared and added to the complete medium. The experimental medium was added to the apical compartment and contained 0 µM (control), 2 µM, 20 µM, 200 µM, or 400 µM quercetin, added to 0.1% DMSO. The normal medium was used in the basal compartment. TEER was measured directly before the addition of quercetin and after 4 h and 24 h. For each condition, three filters were used and the whole setup was repeated 5 times.

### 4.6. Statistical Analysis

Statistical analysis and the plotting of graphics were performed with JMP Pro 15 (Cary, NC, USA) or MS Office 2016 (Redmond, WA, USA). Before the analysis, outliers were identified with Grubb’s test in GraphPad. Statistical testing was performed with a one-way ANOVA and Dunnett’s test for multiple comparisons of TEER and flux data for the incubation experiment. The starting TEER and flux values before quercetin addition, qRT-PCR results, and the densitometry of proteins were analyzed with an unpaired Student’s *t*-test. Values below *p* = 0.05 were considered to be statistically significant. All data are expressed as the mean ± standard error of the mean (SEM) with n being the number of animals or replicates provided for each set of studies. 

## Figures and Tables

**Table 1:** Antibodies used in immunoblotting.

Species	Target	Dilution	Denaturation	Clone	Company
m	Claudin 1	1 µg/mL	95 °C	Polyclonal	Thermo Fisher, Rockford, IL, USA
m	Claudin 2	0.5 µg/mL	95 °C	12H12	Thermo Fisher, Rockford, IL, USA
m	Claudin 2	0.5 µg/mL	9 M Urea, 55 °C	12H12	Thermo Fisher, Rockford, IL, USA
rb	Claudin 4	1 µg/mL	95 °C	3E2C1	Thermo Fisher, Rockford, IL, USA

**Table 2:** Details of the primers used.

Gene	Primer Sequence	Amplicon Length (bp)
Beta Actin (*Sus scrofa*)	(S) 5′-tct ggc acc aca cct tct -3′	114
	(AS) 5′- tga tct ggg tca tct tct cac -3′	
Claudin 1 (*Sus scrofa*)	(S) 5′-tgg aag atg atg agg tgc ag -3′	87
	(AS) 5′-tgg caa cta aga tag cca gac -3′	
Claudin 2 (*Sus scrofa*)	(S) 5′- gca ctg gca tca ccc agt gt -3′	119
	(AS) 5′- gat gat aca ggc caa cga gg -3′	
Claudin 4 (*Sus scrofa*)	(S) 5′- caa ctg cgt gga tga tga ga -3′	140
	(AS) 5′- cca ggg gat tgt aga agt cg -3′	
GAPDH (*Sus scrofa*)	(S) 5′- act cac tct tct acc ttt gat gct -3′	100
	(AS) 5′- tgt tgc tgt agc caa att ca -3′	
YWAHZ (*Sus scrofa*)	(S) 5′- atg caa cca aca cat cct atc -3′	178
	(AS) 5′- gca tta tta gcg tgc tgt ctt -3′	

## Data Availability

Data are contained within the article. The datasets analyzed during the current study are available from the corresponding author upon reasonable request.

## Supplementary Materials

The supporting information can be downloaded at: https://www.mdpi.com/article/10.3390/ijms25031530/s1.
