# Supplementary material for: Quercetin Improves Barrier Properties in Porcine Small Intestine but Not in Peyer’s Patches
Source: Int J Mol Sci. 2024 Jan 26;25(3):1530. doi: 10.3390/ijms25031530 (PMC10855467; doi:10.3390/ijms25031530)
Supplement: Supplementary file 1 [file ijms-25-01530-s001.zip › ijms-2811814-supplementary.pdf]

Table S1. Normalized fold expression of different tight junction protein mRNA levels after 4h incubation with 400 µM quercetin (n = 4, * $p < 0.05$).

gene	tissue	normalized fold expression (mean ± SEM)	p-value
claudin 1	PP	0.81 ± 0.28	0.53
	VE	1.40 ± 0.76	0.61
claudin 2	PP	0.97 ± 0.26	0.91
	VE	0.75 ± 0.18	0.22
claudin 4	PP	1.24 ± 0.52	0.66
	VE	1.7 ± 0.26	0.037 *

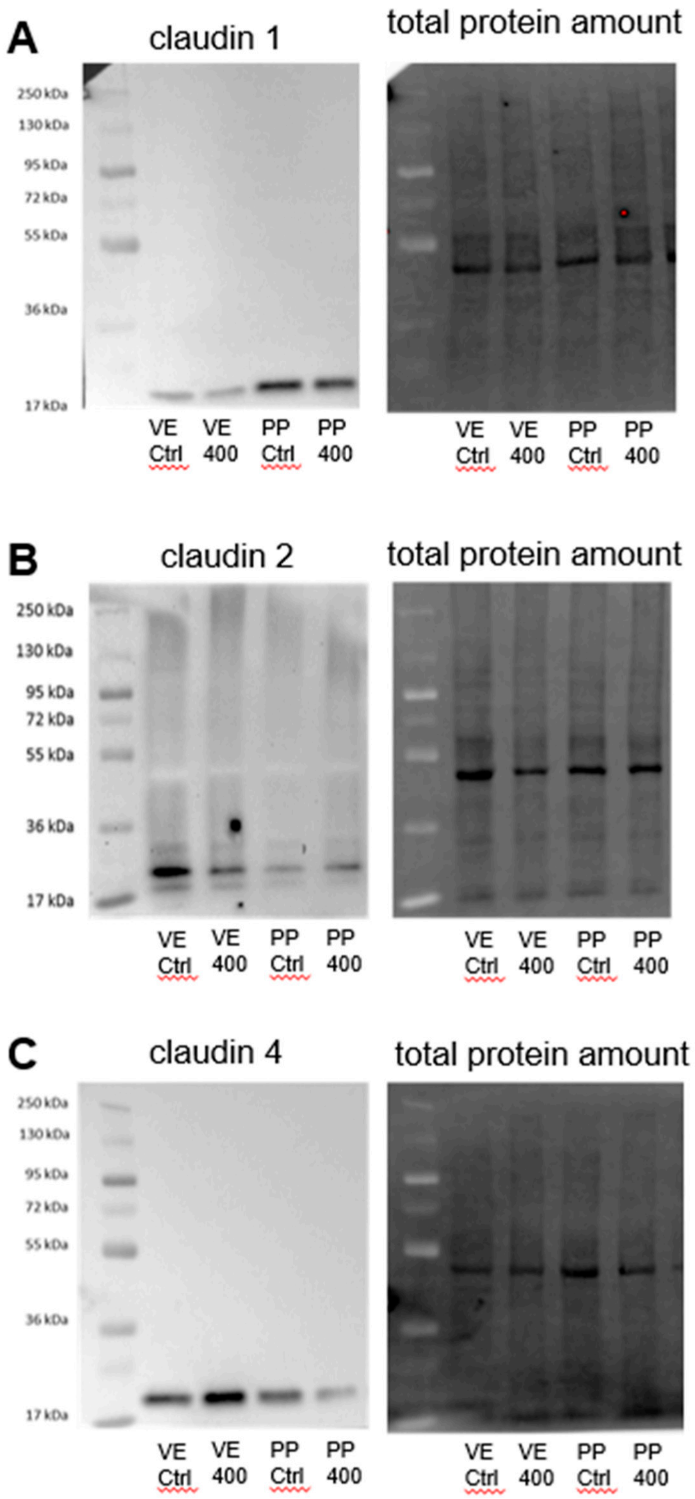

Figure S1. Quantitative protein analysis: PVDF membranes after detection of the total protein amount and the visualized protein bands. Proteins were extracted after 4h of incubation with quercetin Representative membranes were chosen.
